# Exploring nanoarchitectonics and optical properties of PAA-ZnO@BCP wide-band-gap organic semiconductors

**DOI:** 10.1038/s41598-024-53469-3

**Published:** 2024-02-06

**Authors:** A. M. Mansour, Ali B. Abou Hammad, Amany M. El Nahrawy

**Affiliations:** Solid State Physics Department, Physics Research Institute, National Research Centre, 33 El Bohouth St., Dokki, Cairo, 12622 Egypt

**Keywords:** Materials science, Condensed-matter physics, Materials for optics, Nanoscale materials, Structural materials

## Abstract

This work reports the formation of polyacrylic acid (PAA)—zinc oxide (ZnO)—bromocresol purple (BCP), (PAA-ZnO@ (0.00–0.01) BCP wide-bandgap organic semiconductors deposited onto glass substrates via a sol–gel polymerization process. These semiconductor films were deposited on glass substrates using a spin coating and then dried at 60 °C. The PAA-ZnO film appeared to be of amorphous phase, and films loaded with BCP revealed semicrystalline behavior. The surface of the films exhibited adherence and extended grains. The hydrogen bonds formed between PAA-ZnO and the BCP dye within the PAA-ZnO@BCP films was performed using FTIR-spectroscopy. The prepared nanocomposites demonstrate an indirect band transition which is affected slightly by adding ZnO and BCP dye. Optical parameters such as the absorption coefficient, the refractive index, the dielectric constant, optical conductivity, optical depth, and optical electronegativity of the prepared nanocomposites were studied as functions of incident light energy (wavelength). The PAA carbonyl group n-π* transition and BCP aromatic ring π-π* transitions were detected at about 285 (for all samples) and 432 nm (for BCP loaded samples), respectively. The superior photoluminescence characteristics observed in the BCP/PAA-Zn films excited with a wavelength of 250 nm indicated the successful loading of the BCP dye during the self-aggregation of the PAA-Zn film.

## Introduction

Polymers mixing with nanoparticles are interesting for researchers due to their new properties, which are generated by the nanosized effect of nanoparticles. Polymer nanocomposites of different sizes, shapes, and concentrations of nanoparticles are used in many potential optical, electrical, and optoelectronic applications^1,2^. Many synthetic approaches have been used to produce nanocomposites^3^. The interest in the research of polymer nanocomposites is growing due to the improvement in electrical, thermal, optical, and mechanical properties and their great potential as highly functional materials. Nanoparticles embedded in a transparent matrix, in particular, have attracted attention as advanced technological materials due to their high transparency, high refractive index, and attractive electrical properties^4,5^. Polymer nanocomposites also have high thermal stability compared to virgin polymers^6,7^. The importance of polymers is primarily because polymers are still considered inexpensive and easy to manufacture as an alternative material. Nanocomposites demonstrate tremendous promise in a wide range of applications, including optoelectronics, vehicles, drugs, detectors, membranes, packaging, space, coatings, glues, medical patient care, and others^2,8^. The incorporation of nanoparticles into the matrix can be achieved through three primary methods: mixing preformed nanoparticles with the matrix, synthesizing the matrix in the presence of nanoparticles, or synthesizing nanoparticles within the matrix itself^4,9,10^.

Among the various polymers studied, polyacrylic acid (PAA) has received considerable attention due to its excellent properties and applications in various fields, such as electrochemistry, electronics, biomedicine, the solid electrolyte in supercapacitors, an inhibitor of effective corrosion, and ecological non-stick coating^11,12^. The chemical stability of nanoparticles in electronic and electrochemical devices has also been reported to be improved by using PAA coatings^13^. These types of composites have been widely used for various applications in solar cells, wastewater treatment, protective coatings, and biomedical and optical devices^11^. It was also noted that PAA can trap heavy metal ions in low concentrations and can be used as chelating agents. Therefore, it can be used to remove toxic heavy metals from water^14^. Incorporating ZnO nanoparticles into polymeric matrices modifies their optical, mechanical, and thermal properties^15–19^. The properties of nanoparticle/polymer nanocomposites are profoundly influenced by two factors: the dispersion of nanoparticles within the matrix and the interfacial interactions generated with each other^20^. These composites offer a distinct advantage by capitalizing on the high surface area of nanoparticles and the mechanical strength of polymers, often displaying synergistic properties^21^.

The compound 5′,5″-dibromo-o-cresolsulfophthalein, commonly referred to as bromocresol purple (BCP), functions as an organic dye primarily utilized as a pH reagent. It undergoes a color change from yellow under alkaline conditions to red under acidic conditions^22^. The remarkable alteration in color occurs as a consequence of the dynamic polarity variation in the adjacent atmosphere and the rehybridization process of the asymmetric-to-asymmetric resonance profile^23^. Consequently, it manifests its absorption prowess when introduced to nonpolar environments^24^. BCP was employed to detect uric acid, xanthine, albumin, and hypoxanthine^25^. For the detection of ammonia across an extensive range of heat and humidity, a polymer film doped with BCP served as an effective gas detector^26^. The development of the l-tyrosine detector involved the utilization of an enhanced BCP carbon electrode^27^. A BCP film produced through thermal evaporation exhibited an optical bandgap of approximately 2 eV. Furthermore, it demonstrated photodiode behavior even in ambient situations^22^.

In this study, an organic semiconductor (PAA-ZnO) loaded with (0.00–0.01 mol%) BCP was produced starting from polyacrylic acid, zinc acetate, and bromocresol purple (BCP) dye as a dopant using the sol–gel polymerization process, as supported semiconductor organic films. The aim of this work was the investigation of the local structure and optical modifications in PAA-ZnO induced by loading of BCP dye to the semiconductor polymeric matrix. The semiconductor's organic PAA-ZnO/BCP films are achieved by sol–gel process, which is a formation method that is clean, high purity, energy-efficient, and does not cause ineffective yield on the physical properties of the films. The microstructure of the films was characterized by X-ray diffraction, transmission, and scanning electron microscopy (TEM-SEM). The optical properties were measured using photoluminescence and ultraviolet–visible (UV–vis) spectroscopy.

## Experimental

### Sample preparation

Polyacrylic acid-ZnO@BCP (PAA-ZnO@ (0.00–0.01) BCP) organic semiconductor films with adequate structure on glass substrates were formed by sol–gel polymerization processes and dried at 60 °C.

A (2 wt.) Polyacrylic acid (PAA, Sigma Aldrich) dissolved in 60 ml of H_2_O, zinc acetate (Showa: Japan; purity 98%) was first dissolved in H_2_O, and ethanol was then added to the PAA solution. For bromocresol purple (BCP) dye-doped films, the required concentrations of BCP were dissolved in a mixture of H_2_O/ethanol before being loaded within a PAA-Zn solution at room temperature. The organic semiconductor/BCP dye thin films were deposited on glass substrates. Films were dried at 60 °C.

### Characterization

X-ray diffraction (XRD) patterns of the films were recorded on an X-ray diffractometer (XRD-D8 Discover with GADDS Bruker (AXS)) over the range of 5–80° at room temperature. The surface morphology of the deposited films was observed with a scanning electron microscope (TESCAN: VEGA3). High-resolution Transmission Electron Microscope images were possessed using (HRTEM-JEOL/JEM 2100) by (LaB6) source at 200 kV accelerating voltage. FTIR spectra of films nanocomposites, were verified with an FTIR spectrometer (Nicolet Impact:400 FTIR spectrophotometer) in the range of 400–4000 cm^−1^. Normal transmittance and reflectance spectra were recorded using a Jasco (V-570 UV–Vis-NIR) double-beam optical spectrophotometer in the range of 200 to 2500 nm. The reflectance measurement, a 60 mm UV–visible/NIR integrating sphere includes built-in detectors for optimal sensitivity–PMT for UV–visible, InGaAs up to 1600 nm, and PbS up to 2500 nm. Transmittance measurement is performed against a bare glass substrate. The photoluminescence characteristics were assessed using the JASCO Spectrometer/Data System, with evaluations conducted at ExBW 10 and 970 nm.

## Results and discussion

### X-ray diffraction

**Figure 1:**
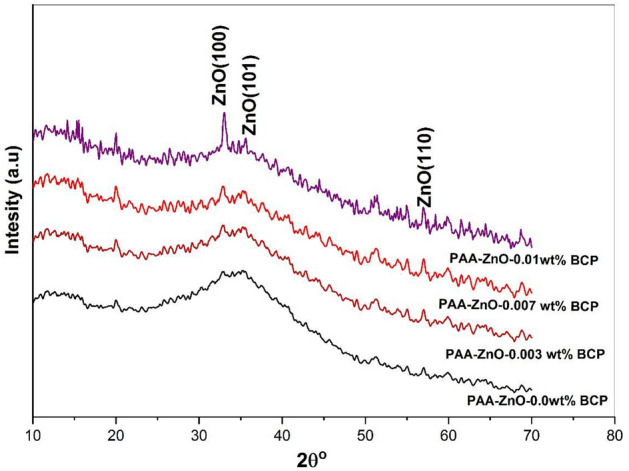
XRD patterns of PAA-ZnO film loaded with different concentrations of BCP.

The structural demeanor of the PAA-organic complex with Zn^2+^ and BCP dye has been investigated using the XRD analysis. Figure 1 shows the XRD pattern of films prepared with x = 0, 0.003, 0.007, and 0.01 wt% BCP (the raw data were attached as supplementary materials sm0, sm0.003, sm0.007 and sm0.01). The patterns reveal a semicrystalline demeanor that is attributed to the polymeric PAA matrix and a lower level of calcination of the compounds made up of structural organic ZnO nanoparticles. According to the limited calcination time at 60 °C, the films were seen to show that the ZnO phase had partially formed, showing the dominance of an amorphous phase and a slight amount of ZnO. The hump at 2θ = 28–40° and the diffraction peaks from 30° up to 60° are for ZnO that can be indexed to hexagonal ZnO (No. 003-0891), showing its partial crystallinity. PAA-ZnO XRD showed that BCP loading increases the degree of crystallinity^12^. The appearance of weak peaks as the BCP ratio increases within the PAA-ZnO matrix, coupled with the disappearance of the well-defined ZnO peaks, suggests a structural transformation in the prepared nanocomposite films^28–31^. This transformation arises from the interaction between the Zn–O bonds and the functional groups present in both PAA and the BCP mixture components^32–34^.

### SEM

**Figure 2:**
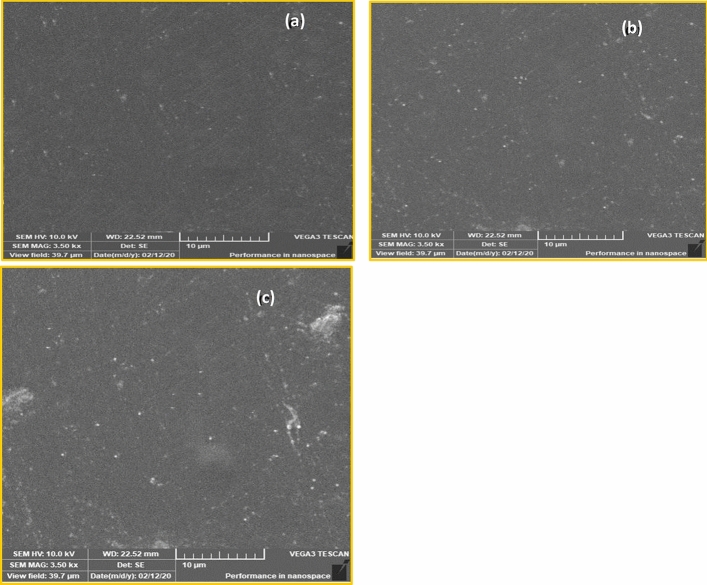
SEM photographs of (**a**) PAA-ZnO composites loaded with BCP (**b**) 0.007 (**c**) 0.01.

Figure 2 presents the surface morphology of PAA-ZnO-BCP through the SEM image. According to the SEM, the surfaces of the films are highly homogeneous, flat, and dense. The SEM images show the presence of dispersed nanospheres on the surface of the samples, and these nanospheres are attributed to ZnO nanoparticles, which are dispersed homogeneously over the surface of the films. According to the XRD investigation, the BCP increases the crystallinity of the ZnO nanoparticles. This behavior also appeared in the SEM image of the film loaded with BCP, where the ZnO nanospheres are observed on the surface of the film loaded with BCP (Fig. 2b,c) than the PAA-ZnO film (Fig. 2a).

### TEM

**Figure 3:**
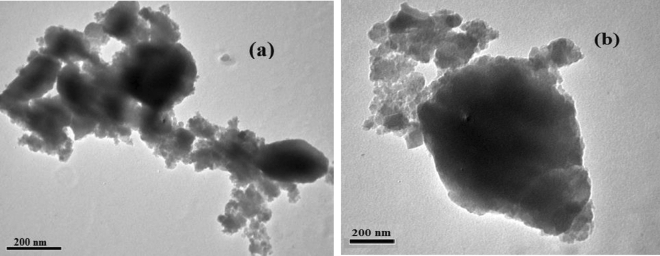
TEM photographs of (**a**) PAA-ZnO composites and (**b**) PAA-ZnO loaded with 0.005 BCP.

TEM image of PAA-ZnO (Fig. 3a and b for PAA-ZnO and PAA-ZnO loaded with 0.005 BCP, respectively) shows the presence of spherical Nps (ZnO-Nps) which are distributed uniformly through the membrane. The dark points in the TEM image result from the accumulation of ZnO-Nps. It is perceived that the accumulation of particles increases with increasing BCP concentrations TEM images confirm the presence of nanospheres with sizes are 10–23 nm, which can be related to ZnO nanoparticles.

### FTIR study

**Figure 4:**
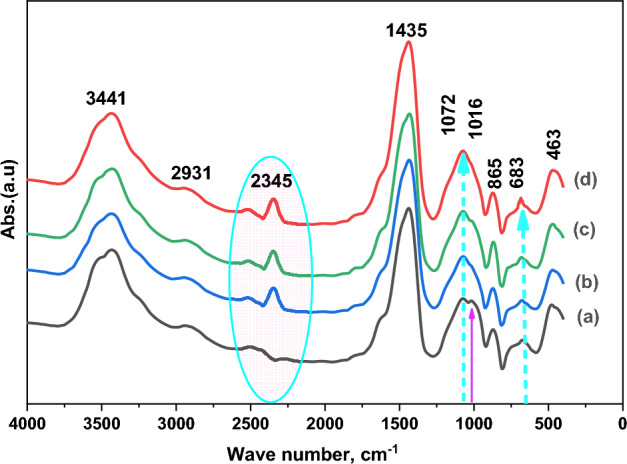
FTIR spectra of (a) PAA-ZnO film loaded with (b) 0.003, (c) 0.005, (d) 0.01 wt% BCP.

FT-IR measurements were conducted to illustrate the interaction between the PAA-ZnO-based nanocomposite film and various ratios (0.00–0.01 wt%) of BCP. Figure 4 exhibits the FTIR spectra of PAA-ZnO, both unloaded and loaded films. It's worth noting that these spectra feature absorption peaks at 3441 cm^−1^ and 2931 cm^−1^, which correspond to the stretching vibrations of hydroxyl groups (OH) and (C–H), respectively^35–37^. Furthermore, it is evident that the bands at 1435 cm^−1^, 1072 cm^−1^, 1016 cm^−1^, and 865 cm^−1^ correspond to the absorption bands associated with the stretching vibrations of (–OH), (C=O), and –CH_2_ in the PAA-ZnO@ BCP films. Additionally, in the loaded PAA-ZnO films, a new bond at 2345 cm^−1^ emerged, while the bond at 1016 cm^−1^ disappeared. These observations confirm the significant influence of BCP loading on the molecular structure of the PAA-ZnO film^29,38,39^.

The bands at 463 cm^−1^ and 683 cm^−1^ can be attributed to the Zn–O bonds, specifically the bending vibration of the Zn–O–Zn bonds within the PAA-ZnO structure. Their intensity undergoes relative changes upon BCP loading^30,31^. Additionally, the absorption peak at 1072 cm^−1^ is a result of the symmetric stretching vibration of the Zn–O bond^30,31^.

Therefore, the alterations in bond intensities in PAA-ZnO@BCP nanocomposite films following BCP loading are indicative of changes in electron density. This, in turn, leads to an enhancement in the optical and photoluminescent properties of the PAA-ZnO@BCP nanocomposite semiconductor films.

### UV–vis spectroscopy

**Figure 5:**
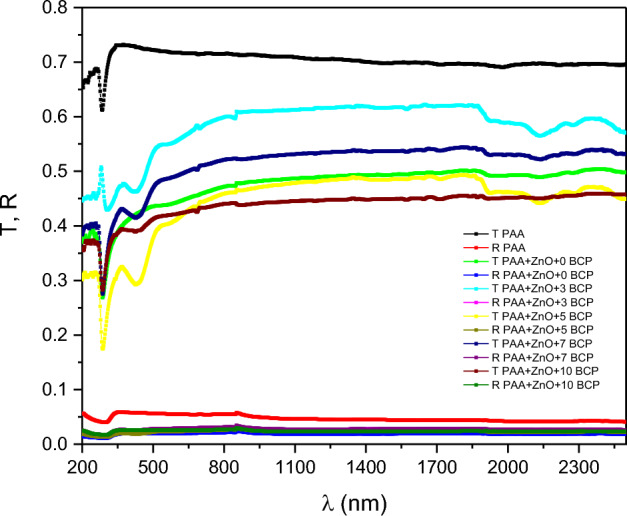
Transmission and reflection spectra of the prepared nanocomposites.

The main task of UV–vis is to identify the properties of optical and electronic materials and to characterize absorption, transmission, and reflection materials. UV–vis absorption can be analyzed when a light beam passes through the sample and is reflected from the surface of the sample. UV absorption is formed on a single wavelength or a wide range of spectral wavelengths. During the transition of the electrons, the excitation takes place at higher energy levels. UV–vis identifies the qualitative and quantitative properties of the sample under test^40^. The direct and indirect bandgap types of optical transitions occur due to the interaction between the incident photons and the valance electrons. However, in the direct bandgap type, there exists a vertical transition profile of the valence electrons to the conduction band^41^. However, the indirect bandgap involves simultaneous interaction with lattice vibrations. At the transition from direct to direct, the electron moment stabilized while the energy was conserved. In the other transition type, i.e. indirect type, the moment of the electron suffering a change^42^. Calculating the absorption coefficient (α) is done by, $I={I}_{0}{\text{exp}}(-\alpha x)$, where $\alpha =2.303/x$, and hence, ${\text{log}}\left(I/{I}_{0}\right)=\left(2.303/x\right)A$. In the case of the direct bandgap, the absorption coefficient is given by, $\alpha h\nu =c{(h\nu -{E}_{g})}^{1/2}$^43^, where c represents a constant related to the sample structure, α represents the absorption coefficient and h is the plank constant. In the case of indirect transition, the absorption coefficient is obtained by, $\alpha h\nu =c{(h\nu -{E}_{g})}^{2}$^43^,

Figure 5 represents the transmission and reflection spectra of the prepared nanocomposites (the raw data were attached as supplementary materials sm PAA(T&R), sm0(T&R), sm0.03(T&R), sm0.05(T&R), sm0.07(T&R), and sm0.01(T&R)). The figure shows a high transmittance of the tested samples that increases with increasing wavelength. The saturation state is reached at about 350 nm for pure PAA sample and that loaded with ZnO. For the samples that are doped with organic dye, the transmittance saturation state is reached at about 525 nm. It was also noted that the transmittance intensity is decreased by adding ZnO as reported before by Singh et al.^44^ and then shows an alternating change by BCP organic dye. On the other hand, the corresponding reflectance of all samples is very small in the same range as the wavelength. These changes in saturation limits and intensity confirm the grafting of organic ZnO and BSP dye on the PAA polymer matrix^45^.

**Figure 6:**
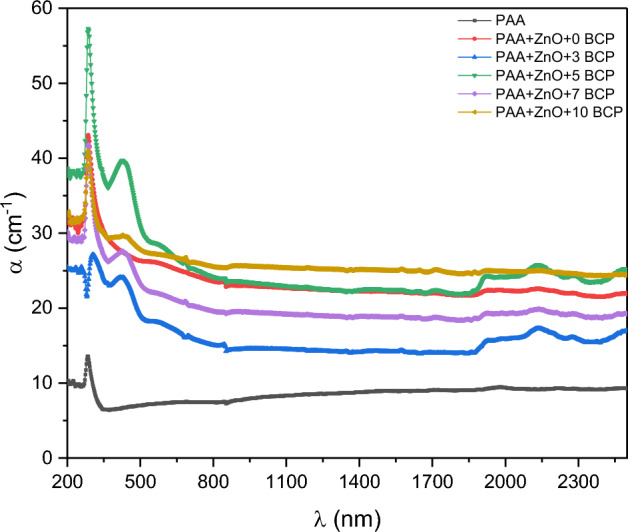
The UV–Vis absorption spectra of the prepared nanocomposites.

The UV–vis absorption spectra of the prepared nanocomposites are presented in Fig. 6. The absorption coefficient shows an absorption peak at about 285 nm for the pure sample (PAA). This peak in the same position is also found in the absorption spectra of the PAA loaded with ZnO and those doped with BCP organic dye. This absorption peak is attributed to the n-π* transition of the carbonyl group in the PAA molecule^46^. Samples doped with organic BCP dye show a second absorption peak at about 423 nm for all doping concentrations and are assigned to π-π* transitions of the aromatic rings of the BCP molecule^22,23,41^.

Davis and Mott expression^47^ for the absorption coefficient as a function of photon energy, $\alpha \left(v\right)={\alpha }_{0}{(h\nu -{E}_{g}^{opt})}^{n}/h\nu$, for both direct and indirect optical transitions, was used to estimate the type and value of the band gap. In this expression, the exponent n = 1/2 for the allowed direct transition and n = 2 for the allowed indirect transition^48^. Plotting of (αhν)^2^ versus photon energy (hν) yields a curve with a straight portion that has an intercept with an x-axis which is equal to the optical energy bandgap.

**Figure 7:**
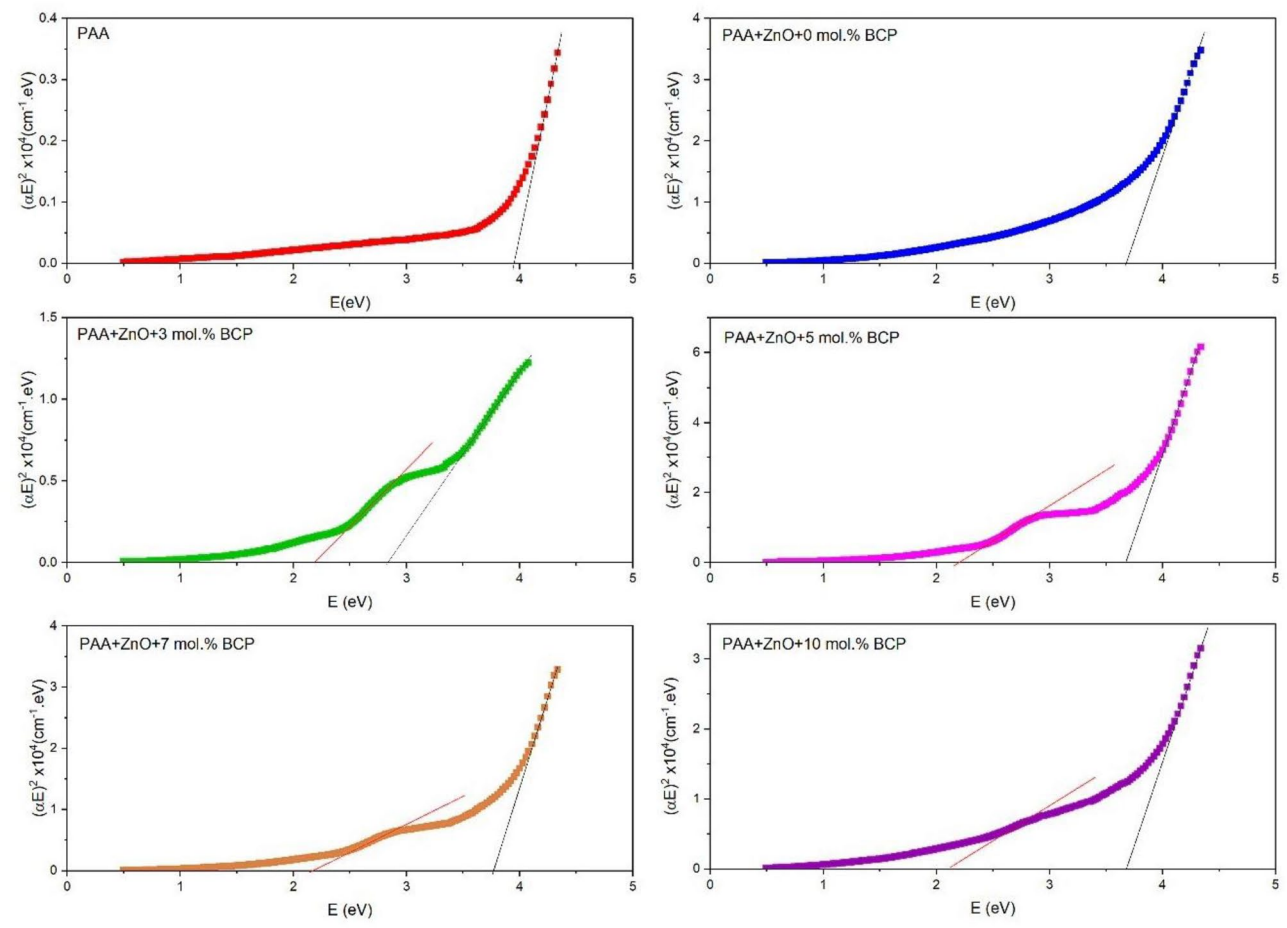
The respective values of ${E}_{g}^{opt}$ is obtained by extrapolating to (αhν)^2^ to 0 for indirect transitions.

**Figure 8:**
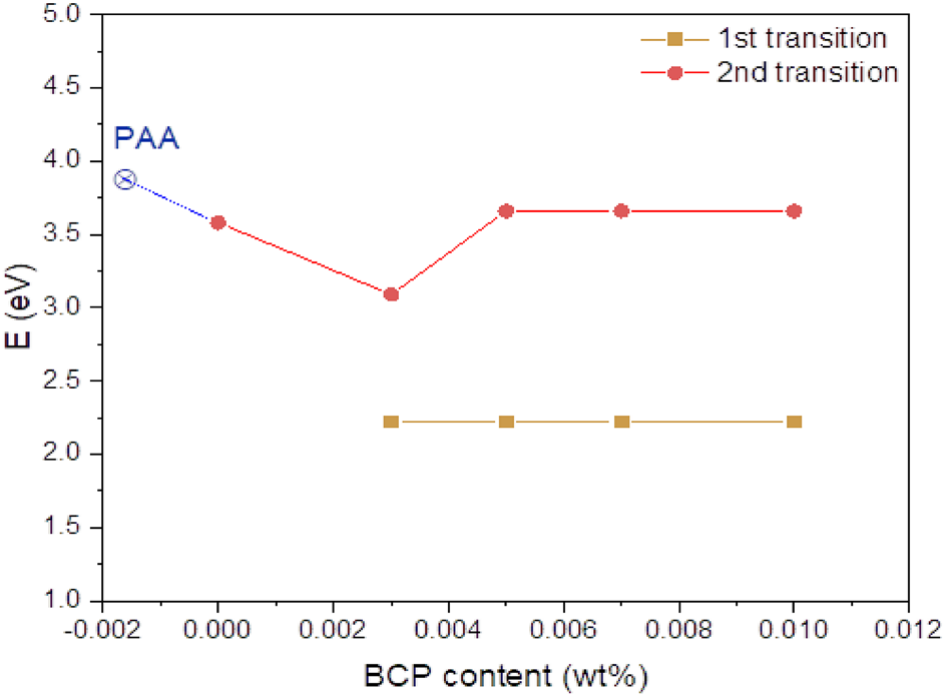
Change of bandgap with change of composition.

The respective values of ${E}_{g}^{opt}$ is obtained by extrapolating to (αhν)^2^ to 0 for indirect transitions as shown in Fig. 7 for all synthesized nanocomposites, and Fig. 8 summarizes the change in band gap with the change of composition. The goodness of fit of the data to the formula for n = 1/2 is determined by the square of the correlation coefficient (R^2^ = 1 is for the perfect fit) which was approximately 0.9995. The error for bandgap energies is ± 0.003 eV. The bandgap value of PAA was found equal to 3.88 eV, which is attributed to the n-π* transition of the carbonyl group in the PAA molecule and it is in agreement with that reported before^46,49^. After loading the PAA matrix with ZnO, the band gap decreases to 3.59 eV, which is similar to that reported by Singh et al. for polyaniline/ZnO nanocomposites^50^. By adding BCP dye, the main bandgap of the composite is slightly decreased to 3.11 eV as found by Hussain et al.^51^ for BCP dye-doped PMMA films. When the concentration of BCP organic dye in the composite is increased slightly to 3.61 and then remains unchanged with further increase. Mansour et al.^23^ reported that the bandgap of BCP films did not change with the increase in the BCP concentration. Furthermore, it was observed that by BCP doping, the second transition in lower energy was generated and assigned to the π-π* transitions of the aromatic rings of BCP^22,23^. This later transition energy did not change with an increase in BCP concentration as was reported before by Mansour et al.^23^.

**Figure 9:**
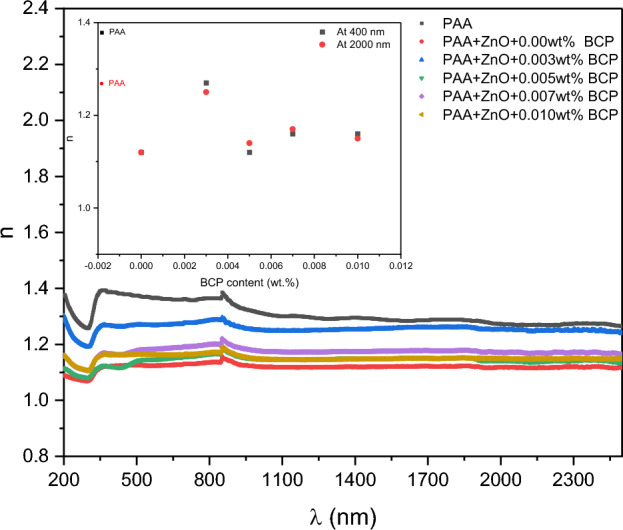
Variation of the refractive index for prepared nanocomposites with the change of incident photon wavelength. The inset represents the change in refractive index with the change in the composition of the samples at low and high wavelengths.

Figure 9 shows the variation of the refractive index for the prepared nanocomposites with the change in incident photon wavelength. The figure shows that the change of the refractive index with the wavelength change is very small and can be considered a constant for all samples. The inset of Fig. 9 represents the change in refractive index with the change in the composition of the samples at low and high wavelengths. It is observed that the change is very small for all nanocomposites where the pure sample (PAA) shows a variation of about 0.1 between the low and high wavelengths, while the other composites show nearly no change. This refractive index behavior may be due to the change in the density of the nanocomposites with the change in the dopant concentration^52^. The nanocomposites with these candidates can be used in applications that need a constant refractive index, as inexpensive and easily prepared materials^53^.

**Figure 10:**
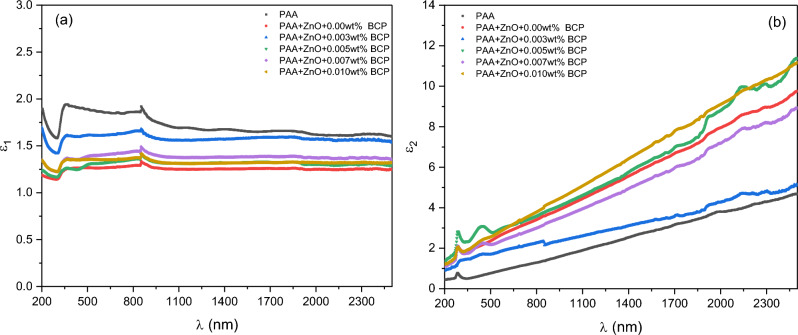
The variation of real and imaginary parts of dielectric constants of the prepared nanocomposites with the photon wavelength.

Figure 10 shows the variation of real and imaginary parts of the dielectric constants of prepared nanocomposites with the photon wavelength. The real part of the dielectric constant (Fig. 10a) shows a nearly stable behavior with an increase in wavelength for all samples. Also, the imaginary part of the dielectric constant (Fig. 10b) shows an increase with the increase of wavelength for all samples. Both real and imaginary parts show a change with a composition change which is a result of the change in the absorption coefficient and refractive index of nanocomposites^54^.

The optical conductivity σ_op_ of the prepared nanocomposites was obtained through^40,55^: ${\sigma }_{op}=\alpha nc{(4\pi )}^{-1}$, where c, α, and n are signs of the velocity of light, the coefficient of absorption and n the refractive index, respectively.

**Figure 11:**
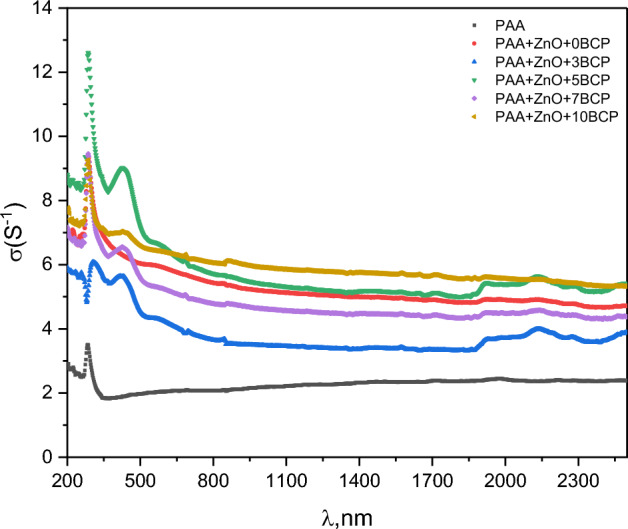
The optical conductivity change of the prepared nanocomposites with the change in light wavelength.

Figure 11 represents the optical conductivity change of the prepared nanocomposites with the change in the light wavelength. The figure shows a height peak at about 285 nm for all samples. This peak is attributed to the n-π* transition of the carbonyl group in PAA molecule^46^, where optical absorption in this region leads to the charge carrier transition, which in turn increases the conductivity. Another height peak is observed only for BCP-loaded samples at a wavelength of about 423 nm. This height peak is assigned to π-π* transitions of the aromatic rings of the BCP molecule^22,23,41^, leading to an increase in conductivity in this region.

**Figure 12:**
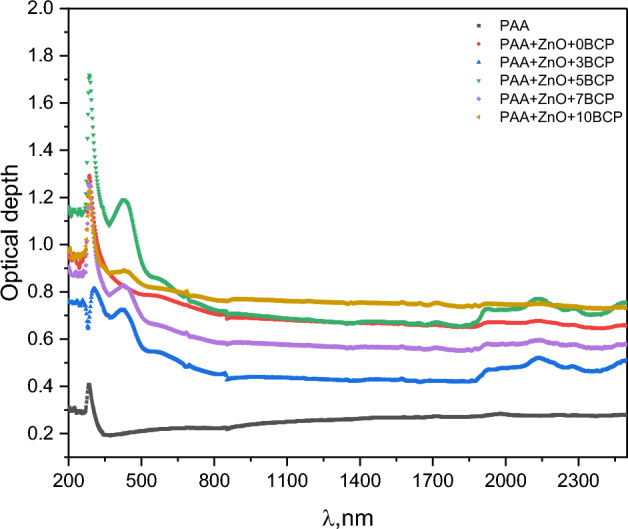
The change in optical conductivity with the change in wavelength of the prepared nanocomposites.

The change in optical conductivity is related to the optical depth of the prepared nanocomposites, which is presented in Fig. 12. The optical depth shows a behavior similar to both the absorption coefficient and optical conductivity. There are two peaks at about 285 nm (for all samples) and 423 nm (observed only for BCP-loaded samples). These peaks are related to the n-π* transition of the carbonyl group in the PAA molecule^46^, and π-π* transitions of the aromatic rings of the BCP molecule^22,23,41^, respectively.

The optical electronegativity (μ) is known as the atomic tendency to gain electrons from the anionic band. The refractive index n is used to estimate the optical electronegativity of the prepared nanocomposites through^22^: ${\eta }_{op}={(A/n)}^{0.25}$ where A is a dimensionless constant.

**Figure 13:**
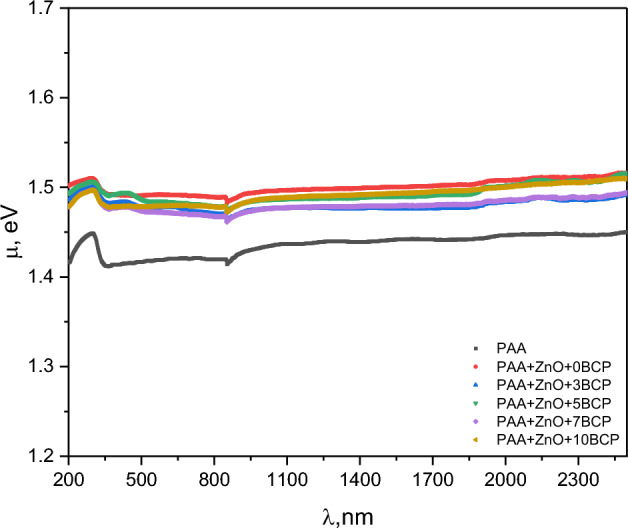
The optical electronegativity with the change of wavelength of the prepared nanocomposites.

The optical electronegativity is nearly unchanged with incident light wavelength change for all samples, Fig. 13. Also, it was noted that the change due to compositions is very small and is related to the change of absorption and optical depth of the nanocomposites.

### Effect of BCP dose on PL

**Figure 14:**
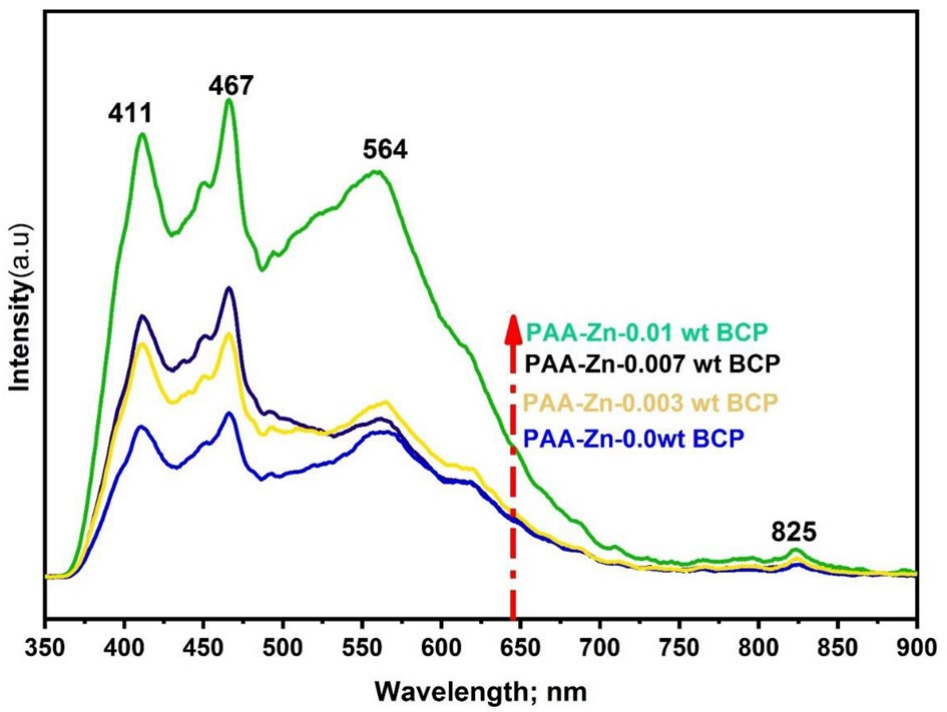
Photoluminescence spectra of PAA-ZnO film and load with different (0.003–0.01 wt%) of BCP dye.

The effect of BCP dose on the photoluminescence of PAA-ZnO nanocomposite on glass substrate and excited at 250 nm is presented in Fig. 14 (the raw data were attached as supplementary materials smPL0.0, smPL0.003, smPL0.007, and smPL0.01). With increasing the BCP from 0.003 to 0.01 wt% the Photoluminescence of PAA-ZnO nanocomposite increased within the range from 411 to 824 nm. This trend is related to the formation of a larger number of binding sites for BCP in the PAA-ZnO nanocomposite.

The BCP/PAA-ZnO films display absorption peaks within the 411–824 nm range, characterized by weak and broad spectral shapes. The primary absorption peaks in the BCP spectra occur at 411 nm, 467 nm, and 564 nm. These peaks are attributed to the intra-transitions originating from the loading of BCP within the BCP/PAA-ZnO complex. Figure 14 reveals a distinct overlap between the photoluminescence of PAA-ZnO and the absorption of the BCP dye, indicating a favorable condition for efficient energy transfer from the PAA-Zn nanocomposite to the dye molecule through the Förster mechanism^56^.

The enhancement of the three primary peaks can be attributed to the increased BCP content, which is associated with BCP's possession of p-p conjugated bonds, a larger real surface area, higher conductivity, and a greater number of active sites^57^.

Additionally, the change in the main peaks (411–564 nm) can indicate that there was a successful interaction between BCP and PAA-Zn complex during the film formation. There is a relative change at higher energy (> 700 nm), which may be attributed to the lower conjugation of the BCP due to the carrier recombination occurring.

## Conclusions

BCP dye was effectively loaded using sol–gel polymerization within PAA-ZnO nanocomposites. The XRD of PAA-ZnO films shows a lower crystallinity degree determined by the locally ordered arrangement of polymeric chains and gradually increases after the addition of BCP. The SEM with TEM confirms the compatibility between the ZnO-Nps and PAA Polymer, therefore they can be used to form a controllable hybrid nanocomposite membrane. An optical absorption peaks related to the n-π* transition of the carbonyl group in the PAA molecule and π-π* transitions of the aromatic rings of the BCP were detected at about 285 (for all samples) and 432 nm (for BCP loaded samples). The refractive index, the dielectric constant, optical conductivity, optical depth, and optical electronegativity of the prepared nanocomposites were extracted. The indirect band transition was displayed for the nanocomposites. The loading of ZnO and BCP in PAA is an essential factor that affects the crystalline degree, optical, surface morphology, and quality of PAA-ZnO/BCP films. So-gel polymerization enables short reaction times, enhanced product purities, reduced contaminating reactions, and high energy efficiency, hence, an effective reaction rate compared with the conventional method. This method allows for precise process control and ensures a uniform distribution of BCP within the nanocomposite films. With increasing of BCP contents, the BCP/PAA-ZnO films exhibited remarkable photoluminescence behavior. The examination of PAA-ZnO@BCP nanocomposite films, characterized by their wide band gap and impressive photoluminescence, introduces a novel approach to enhance the spectroscopic attributes of PAA-ZnO-based nanocomposite films. This development holds significant potential for advancing optoelectronic devices, such as solar cells, supercapacitors, photodetectors, and photoluminescence devices.

### Supplementary Information

Supplementary Information 1.

Supplementary Information 2.

Supplementary Information 3.

Supplementary Information 4.

Supplementary Information 5.

Supplementary Information 6.

Supplementary Information 7.

Supplementary Information 8.

Supplementary Information 9.

Supplementary Information 10.

Supplementary Information 11.

Supplementary Information 12.

Supplementary Information 13.

Supplementary Information 14.

Supplementary Information 15.

Supplementary Information 16.

Supplementary Information 17.

Supplementary Information 18.

Supplementary Information 19.

Supplementary Information 20.

## Data Availability

All data generated or analyzed during this study are included in this published article and are also available as supplementary materials.

## References

[1] Abd-Elnaiem AM, Rashad M, Hanafy TA, Shaalan NM (2023). Improvement of optical properties of functionalized polyvinyl alcohol-zinc oxide hybrid nanocomposites for wide UV optoelectronic applications. J. Inorg. Organomet. Polym. Mater..

[2] Hussain F, Hojjati M, Okamoto M, Gorga RE (2006). Review article: Polymer-matrix nanocomposites, processing, manufacturing, and application: An overview. J. Compos. Mater..

[3] Krishnan PMG, Sobha A, Balakrishnan MP, Sumangala R (2014). Synthesis and characterization of Ag/PVP nanocomposites by reduction method. OAlib.

[4] Higazy AA, Afifi H, Khafagy AH, El-Shahawy MA, Mansour AM (2006). Ultrasonic studies on polystyrene/styrene butadiene rubber polymer blends filled with glass fiber and talc. Ultrasonics.

[5] Xiao G (2015). Visible-light-mediated synergistic photocatalytic antimicrobial effects and mechanism of Ag-nanoparticles@chitosan–TiO_2_ organic–inorganic composites for water disinfection. Appl. Catal. B.

[6] Chandrakala H, Somashekarappa H, Somashekar R, Chinmayee S (2014). Poly(vinyl alcohol)/zincoxide-ceriumoxide nanocomposites: Electrical, optical, structural and morphological characteristics. Indian J. Adv. Chem. Sci..

[7] Khan WS, Hamadneh NN, Khan WA, Di Sia P (2016). Polymer nanocomposites–synthesis techniques, classification and properties. Science and Applications of Tailored Nanostructures.

[8] Sonawane, G. H., Patil, S. P. & Sonawane, S. H. Nanocomposites and its applications. In *Applications of Nanomaterials* 1–22 (Elsevier, 2018) 10.1016/b978-0-08-101971-9.00001-6.

[9] El Nahrawy AM, Montaser AS, Bakr AM, Abou Hammad AB, Mansour AM (2021). Impact of ZnO on the spectroscopic, mechanical, and UPF properties of Fe_2_O_3_-tough polystyrene-based nanocomposites. J. Mater. Sci. Mater. Electron..

[10] El Nahrawy AM, Hammad ABA, Youssef AM, Mansour AM, Othman AM (2019). Thermal, dielectric and antimicrobial properties of polystyrene-assisted/ITO: Cu nanocomposites. Appl. Phys. A Mater. Sci. Process.

[11] Shaik MR (2016). Modified polyacrylic acid-zinc composites: Synthesis, characterization and biological activity. Molecules.

[12] Sözügeçer S, Bayramgil NP (2021). Preparation and characterization of polyacrylic acid-hydroxyapatite nanocomposite by microwave-assisted synthesis method. Heliyon.

[13] Wang W (2022). Highly sensitive and selective surface acoustic wave ammonia sensor operated at room temperature with a polyacrylic acid sensing layer. Sensors.

[14] Shahid SA, Qidwai AA, Anwar F, Ullah I, Rashid U (2012). Improvement in the water retention characteristics of sandy loam soil using a newly synthesized poly(acrylamide-co-acrylic acid)/AlZnFe_2_O_4_ superabsorbent hydrogel nanocomposite material. Molecules.

[15] Davar F, Majedi A, Mirzaei A (2015). Green synthesis of ZnO nanoparticles and its application in the degradation of some dyes. J. Am. Ceram. Soc..

[16] Kumar S, Boro JC, Ray D, Mukherjee A, Dutta J (2019). Bionanocomposite films of agar incorporated with ZnO nanoparticles as an active packaging material for shelf life extension of green grape. Heliyon.

[17] Shahmohammadi Jebel F, Almasi H (2016). Morphological, physical, antimicrobial and release properties of ZnO nanoparticles-loaded bacterial cellulose films. Carbohydr. Polym..

[18] Shankar S, Teng X, Li G, Rhim JW (2015). Preparation, characterization, and antimicrobial activity of gelatin/ZnO nanocomposite films. Food Hydrocoll..

[19] Gomes ATA (2011). Le criquet nomade révèle son code couleur. Int. J. Biomed. Imaging.

[20] El Nahrawy AM (2022). Talented Bi_0.5_Na_0.25_K_0.25_TiO_3_/oxidized cellulose films for optoelectronic and bioburden of pathogenic microbes. Carbohydr. Polym..

[21] Abou Hammad AB, Mansour AM, Elhelali TM, El Nahrawy AM (2023). Sol-Gel/Gel casting nanoarchitectonics of hybrid Fe_2_O_3_–ZnO/PS-PEG nanocomposites and their optomagnetic properties. J. Inorg. Organomet. Polym. Mater..

[22] Mansour AM (2019). Fabrication and characterization of a photodiode based on 5′,5′′-dibromo-o-cresolsulfophthalein (BCP). Silicon.

[23] Mansour AM, Nasr M, Saleh HA, Mahmoud GM (2019). Physical characterization of 5′,5″-dibromo-o-cresolsulfophthalein (BCP) spin-coated thin films and BCP/p-Si based diode. Appl. Phys. A Mater. Sci. Process.

[24] Walsh CA (1993). Orientational relaxation in electric field poled guest—host and side-chain polymers below Tg. Macromolecules.

[25] Wang Y, Tong LL (2010). Electrochemical sensor for simultaneous determination of uric acid, xanthine and hypoxanthine based on poly (bromocresol purple) modified glassy carbon electrode. Sens. Actuators B Chem..

[26] Choudhury S, Chitra R, Yakhmi JV (2003). Studies on the formation of Langmuir monolayer and Langmuir-Blodgett films of octadecyl amine-bromocresol purple dye complex. Thin Solid Films.

[27] Shrestha S (2016). Amperometric sensor based on multi-walled carbon nanotube and poly (Bromocresol purple) modified carbon paste electrode for the sensitive determination of l-tyrosine in food and biological samples. J. Electroanal. Chem..

[28] Okamoto K, Luscombe CK (2011). Controlled polymerizations for the synthesis of semiconducting conjugated polymers. Polym. Chem..

[29] Tan XM, Rodrigue D (2019). A review on porous polymeric membrane preparation. Part I: Production techniques with polysulfone and poly (vinylidene fluoride). Polymers.

[30] Babaei P, Safaei-Ghomi J (2021). l-proline covered N doped graphene quantum dots modified CuO/ZnO hexagonal nanocomposite as a robust retrievable catalyst in synthesis of substituted chiral 2-amino-4H-chromenes. Mater. Chem. Phys..

[31] Babaei P, Safaei-Ghomi J, Rashki S, Mahmoudi Kharazm A (2023). Morphology modified by polyvinylpyrrolidone for enhanced antibacterial and catalytic execution of bioactive Ag/ZnO composites based on hydroxyapatite in the synthesis of O-Aminocarbonitriles. Ceram. Int..

[32] Abouelnaga AM, Meaz TM, El Nahrawy AM (2021). Expansion of nanosized MgSiO_3_/chitosan nanocomposite structural and spectroscopic for loading velosef by nanomaterial intervention. ECS J. Solid State Sci. Technol..

[33] Youssef AM, El-Nahrawy AM, Abou Hammad AB (2017). Sol-gel synthesis and characterizations of hybrid chitosan-PEG/calcium silicate nanocomposite modified with ZnO-NPs and (E102) for optical and antibacterial applications. Int. J. Biol. Macromol..

[34] El Nahrawy AM, Abou Hammad AB, Bakr AM, Shaheen TI, Mansour AM (2020). Sol–gel synthesis and physical characterization of high impact polystyrene nanocomposites based on Fe_2_O_3_ doped with ZnO. Appl. Phys. A.

[35] El-naggar AM (2023). Exploring the structural, optical and electrical characteristics of PVA/PANi blends. Opt. Mater..

[36] Abdelhamied MM, Atta A, Abdelreheem AM, Farag ATM, El Sherbiny MA (2021). Oxygen ion induced variations in the structural and linear/nonlinear optical properties of the PVA/PANI/Ag nanocomposite film. Inorg. Chem. Commun..

[37] Elnahrawy AM, Kim YS, Ali AI (2016). Synthesis of hybrid chitosan/calcium aluminosilicate using a sol-gel method for optical applications. J. Alloys Compd..

[38] Gupta M, Tomar RS, Kaushik S, Mishra RK, Sharma D (2018). Effective antimicrobial activity of green ZnO nano particles of *Catharanthus roseus*. Front. Microbiol..

[39] Chithra MJ, Pushpanathan K (2016). Thermal, structural and optical investigation of Cu-doped ZnO nanoparticles. Mod. Phys. Lett. B.

[40] El Nahrawy AM, Mansour AM, Abou Hammad AB, Wassel AR (2019). Effect of Cu incorporation on morphology and optical band gap properties of nano-porous lithium magneso-silicate (LMS) thin films. Mater. Res. Express.

[41] Farag AAM, Mansour AM, Ammar AH, Rafea MA (2011). Characterization of electrical and optical absorption of organic based methyl orange for photovoltaic application. Synth. Met..

[42] Farag AAM, Osiris WG, Ammar AH, Mansour AM (2013). Electrical and photosensing performance of heterojunction device based on organic thin film structure. Synth. Met..

[43] Hassan N, Mansour AM, Roushdy N, Farag AAM, Osiris WG (2018). Optical sensing performance characteristics of Schottky devices diodes based nano-particle disodium 6-hydroxy-5-[(2-methoxy-5-methyl-4-sulfophenyl)azo]-2-naphthalenesulfonate thin films: A comparison study. Optik.

[44] Singh DP, Madhav H, Jaiswar G (2016). Effects of zinc oxide on polyacrylic acid: A core-shell. Sci. Eng. Appl..

[45] Abd El-Mageed HR, Abd El-Salam HM, Eissa MF (2018). Spectroscopic study on poly(acrylic acid-coacrylamide)- graft-polyaniline as a radiation dosimeter for alpha particles. Radiat. Prot. Dosim..

[46] El-Diasty F, Aly EH, El-Sawy NM (2010). Optical and two-photon absorption analysis of radiation-grafted fluoropolymer. J. Polym. Sci. B Polym. Phys..

[47] Davis EAA, Mott NFF (1970). Conduction in non-crystalline systems V. Conductivity, optical absorption and photoconductivity in amorphous semiconductors. Philos. Mag..

[48] Hemdan BA, El Nahrawy AM, Mansour AM, Hammad ABA (2019). Green sol–gel synthesis of novel nanoporous copper aluminosilicate for the eradication of pathogenic microbes in drinking water and wastewater treatment. Environ. Sci. Pollut. Res..

[49] Agool IR, Ali M, Hashim A (2014). Polyvinyl alcohol–poly-acrylic acid–titanium nanoparticles nanocomposites: Optical properties. Adv. Nat. Appl. Sci..

[50] Singh R, Choudhary RB (2016). Optical absorbance and ohmic behavior of PANI and PANI/ZnO nanocomposites for solar cell application. Optik.

[51] Badran HA, Al-Fregi AA, Alfahed RKF, Al-Asadi AS (2017). Study of thermal lens technique and third-order nonlinear susceptibility of PMMA base containing 5′,5′′-dibromo-o-cresolsulfophthalein. J. Mater. Sci. Mater. Electron..

[52] Ugwu EI, Ph D (2006). Optical properties of iron halide (FeCl_2_) thin film deposited using solution growth technique (SGT). Pac. J. Sci. Technol.

[53] El Nahrawy AM, Hammad ABA, Youssef AM, Mansour AM, Othman AM (2019). Thermal, dielectric and antimicrobial properties of polystyrene-assisted/ITO: Cu nanocomposites. Appl. Phys. A Mater. Sci. Process.

[54] Nahida JH, Marwa RF (2011). Study of the optical constants of the PMMA/PC blends. AIP Conf. Proc..

[55] El Nahrawy AM (2020). Optical, functional impact and antimicrobial of chitosan/phosphosilicate/Al_2_O_3_ nanosheets. J. Inorg. Organomet. Polym. Mater..

[56] Rai VK, Srivastava R, Kamalasanan MN (2009). White organic light-emitting diodes based on blue fluorescent bis(2-(2-hydroxyphenyl)benzoxazolate)zinc [Zn(hpb)2] doped with DCM dye. Synth. Met..

[57] Zhu X (2015). Green synthesis of a bromocresol purple/graphene composite and its application in electrochemical determination of 2,4,6-trichlorophenol. Anal. Methods.

